# Effect of Current Waveform on Microstructure Evolution and Mechanical Properties of GH4169 High-Temperature Alloy Tungsten Inert Gas Additive Manufacturing

**DOI:** 10.3390/ma17184649

**Published:** 2024-09-22

**Authors:** Xinlong Zhang, Jiaao Zhang, Xiaodong Xie, Zhaosong Jiang, Chao Chen, Zhe Wu, Yang Zhang

**Affiliations:** 1 College of Mechanical and Electrical Engineering, Northeast Forestry University, Harbin 150040, China; zhangxinlong2009@nefu.edu.cn (X.Z.); zja89@nefu.edu.cn (J.Z.); 2022111925@nefu.edu.cn (X.X.); jzs@nefu.edu.cn (Z.J.); wuzhe@nefu.edu.cn (Z.W.); 2 College of Materials Science and Engineering, Jilin University, Changchun 130015, China; chaochenaw@126.com; 3 College of Science, Northeast Forestry University, Harbin 150040, China

**Keywords:** current waveform, GH4169, TIG additive manufacturing, microstructure evolution, mechanical properties

## Abstract

Direct current (DC) and pulsed DC tungsten inert gas (TIG) additive manufacturing processes were employed to fabricate GH4169 high-temperature alloy specimens. Upon comparing and analysing the two additive manufacturing methods, the evolution of microstructure and mechanical properties of the additively manufactured specimens were discussed. It provided a useful reference for the engineering application of pulsed DC TIG technology. The results showed that the overall forming process of the specimen was relatively stable under the DC TIG additive manufacturing and pulsed DC TIG additive manufacturing processes. The aspect ratio of the deposited layer of the pulsed DC-deposited specimen was relatively low, and the deposited layer of the pulsed DC specimen became flatter, which was conducive to maintaining the stability of the molten pool during the deposition process and improving forming accuracy. The microstructure distribution of the deposited layer from bottom to top was relatively uneven, with columnar dendrites in the bottom layer, cellular crystals in the middle layer, and equiaxed crystals in the top layer. Compared with the DC TIG additive manufacturing of GH4169 high-temperature alloy specimens, the Laves phase of the pulsed DC specimens was significantly reduced, which improved the plasticity and brittleness of the material.

## 1. Introduction

The GH4169 high-temperature alloy, due to its good resistance to high temperatures, thermal corrosion, fatigue, oxidation, and crack extension resistance, has excellent high-temperature microstructure stability. The GH4169 high-temperature alloy is widely used in aerospace, nuclear energy, petrochemical, and other fields [1,2]. Among them, the high hardness and low thermal conductivity of the GH4169 high-temperature alloy bring difficulties to the conventional processing and moulding process. The disadvantages, such as high equipment loss, long manufacturing cycles, and high production costs, are highlighted. This is especially true for parts with complex structural shapes and high-precision dimensional requirements [3,4,5].

Wire and arc additive manufacturing (WAAM) has the benefits of high moulding efficiency and low cost and shows an extremely broad application potential in the manufacture of large-sized structural parts. Arc additive manufacturing technology is considered to be the pivotal direction of engineering value in the field of additive manufacturing [6,7]. Arc additive manufacturing technology is divided into metal inert gas (MIG) arc additive manufacturing technology and tungsten inert gas (TIG) arc additive manufacturing technology. The disadvantages of MIG arc additive manufacturing technology, such as producing large spattering, poor side forming accuracy, high cost, and the tendency to produce pollutant gases, have limited the scope of its application. In contrast, the advantages of TIG arc additive manufacturing technology, such as a stable stacking process, small splash, and environmental protection, have greatly promoted its application in the manufacture of large metal parts [8,9]. However, the TIG arc additive manufacturing process parameters are intricate, and there exists a robust interplay between the arc, the melt droplet, and the melt pool. As the degree of heat accumulation increases, the melt pool is prone to instability and collapse, resulting in difficulty in controlling the forming process. This restricts the wide application of TIG in the manufacture of large-sized components with low-cost and high-efficiency forming. Therefore, it is essential that we conduct research into the fundamental processes and principles of TIG arc additive to control its shape and properties, thereby managing the forming process and enhancing its mechanical properties.

Pulsed TIG (PTIG) additive manufacturing belongs to an important branch of arc additive manufacturing technology. Its periodic changes in the arc serve as a heat source, while argon or other inert gases provide protection. The filler wire is deposited drop by drop and layer by layer in the form of molten droplets. This results in near-net-shape fabricated parts [10]. The most significant advantages of PTIG additive manufacturing technology are affordability, rapid deposition rates, and material utilisation. This technology is particularly well-suited for the production of large-scale and intricate structural components [11,12]. Therefore, PTIG additive manufacturing technology in aerospace, aviation, automotive, nuclear power, and other fields of parts manufacturing has a good application prospect [13,14].

The research of PTIG additive manufacturing technology is still in its nascent stages. In terms of materials, scholars have primarily focused their studies on magnesium aluminium alloy [15], nickel-based alloy [16], and Ti-6Al-4V [17,18]. Successful fabrication has been achieved for simple structural components, including thin-walled plates and cylindrical shapes. Guo et al. [19] established a regression model of PTIG process parameters and Inconel 625 single weld channel forming quality. The influence law of the main effect and interaction effect of process parameters on the forming quality of the weld channel was elucidated. Experiments show that the established model can be used for the prediction of forming quality. Ouyang et al. [20] used PTIG additive manufacturing of 5356 aluminium alloy specimens to improve the deposition conditions and reduce the deformation of the formed parts by preheating the substrate, monitoring the arc length, and regulating the welding current. The effects of process parameters on the width and thickness of the deposited layer were also elucidated. The results show that 5356 aluminium alloys with high forming quality can be fabricated by PTIG additive manufacturing. Katou et al. [21] used PTIG additive manufacturing of Ti alloy parts to investigate the relationship between process parameters and forming quality and successfully prepared Ti alloy formed parts. The study pointed out that with the increase of peak current, the inclination and height of the weld channel decreased gradually, and the melt width increased gradually. No defects, such as porosity and cracks, were found in the formed parts, and the microstructure was relatively uniform.

In this experiment, GH4169 high-temperature alloy specimens were fabricated using DC and pulsed DC TIG additive manufacturing. By comparing and analysing the two deposition methods, the evolution law of microstructure and mechanical properties of the deposition and bonding layer are explored with the expectation of providing a useful reference for the engineering application of PTIG technology.

## 2. Experimental Materials and Methods

### 2.1. Test Material

The substrate material used in the TIG additive manufacturing test was Q235 steel, with a substrate size of 200 × 100 × 10 mm, and its chemical composition was taken from the material data sheet (GB/T 700-2006), as shown in Table 1 [22]. In order to reduce the impact of impurities on the surface of the substrate material on weld shaping, it was polished before the experiment, wiped with acetone, and dried. The filler material used in the additive test was GH4169 high-temperature alloy welding wire with a diameter of 1.2 mm, and its chemical composition was determined experimentally, as shown in Table 2. The additive manufactured single layer measured 170 mm in length and deposited 35 layers.

### 2.2. Test Methods

**Figure 1:**
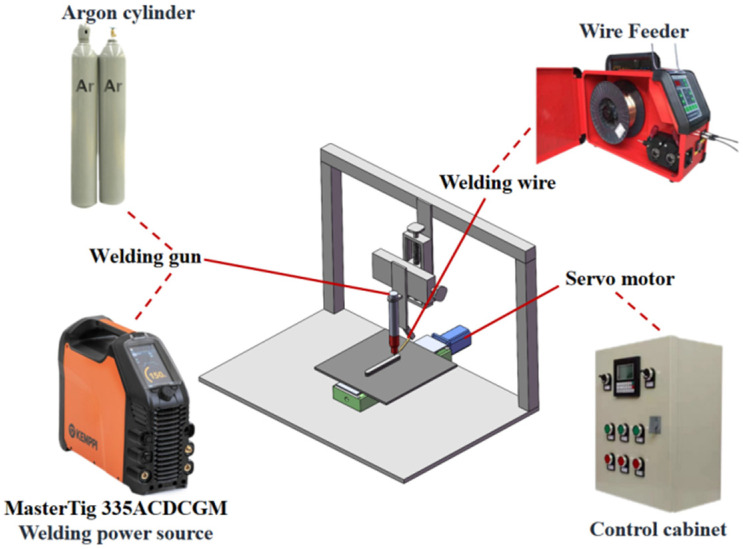
Schematic diagram of the TIG additive manufacturing process.

The arc fuse system used for the test consisted of a welding machine (KEMPPI, MASTER TIG335, Lahti, Finland) and a multifunctional automatic argon arc wire filling machine (WEILD, WF-007A, Guangzhou, China). The utilisation of a reciprocating start and finish arc mode enabled the deposition of a single-pass multilayer specimen. The deposition process of the torch was always kept perpendicular to the substrate. The experimental principle is shown in Figure 1. The High-Speed Image System (Rocketech Technology Corp., Ltd., Acuteye, Changsha, China), which mainly consisted of a high-speed camera, a ruggedised portable industrial controller, a special light source, and an optical lens. The process parameters are detailed in Table 3, where sample A was the GH4169 high-temperature alloy specimen prepared by DC deposition, and sample B was the GH4169 high-temperature alloy specimen prepared by pulsed DC deposition.

**Figure 2:**
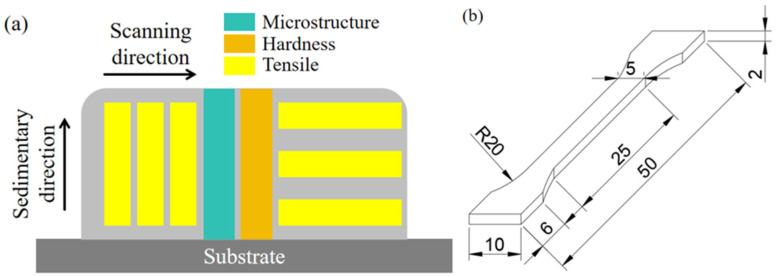
Specimen cutting locations and tensile specimen dimensions. (**a**) Cutting position of the specimen and (**b**) the size of the tensile specimen.

A CNC wire cutting machine (Cyang, DK7740, Dongguan, China) was used to cut the deposited specimens, and the positions of the microtest specimens, hardness test specimens, and tensile test specimens are given in Figure 2a. The microtest specimens and the hardness test specimens were cut along the direction of the cross-section (y-z plane), with a width of 10 mm for both specimens. The tensile test specimen parallel to the printing direction was marked as the horizontal direction, and the tensile specimen parallel to the deposition direction was marked as the vertical direction. The size of the tensile specimen (GB/T 228.1-2021 standard) is shown in Figure 2b [23]. The cut microtest specimens and hardness test specimens were cleaned with acetone solution in an ultrasonic cleaner to remove surface oils and impurities. The specimens were sequentially sanded with 180 to 1500# metallographic sandpaper and polished with a polishing machine. The specimens were etched with an etching solution (anhydrous copper sulphate powder 2 g, anhydrous ethanol 10 mL, and hydrochloric acid 15 mL) for 20 s. Subsequently, the microstructures of the microtest specimens were analysed by optical microscopy (ZEISS, Axio Scope. A1, Oberkochen, Germany), body microscopy (OLYMPUS, SZX7, Tokyo, Japan), and scanning electron microscopy (HITACHI, SU5000, Tokyo, Japan). Measurements of the specimens were analysed using Image-Pro Plus software 6.0 (Media Cybernetics, Rockville, MD, USA). Using a digital microhardness tester (Grows, HVS-1000, Shanghai, China), the hardness test specimens were tested for microhardness according to GB/T 4340.4-2022 [24]. The hitting points were spaced 2.5 mm from top to bottom, the load was 200 g, and the holding time was 15 s. Using a microcomputer-controlled electronic universal testing machine (Kexin, WDW-100, Changchun, China), the tensile test was carried out on the tensile specimen at room temperature, in accordance with GB/T 228.1-2021, and the tensile rate was 1 mm/min [23]. The tensile fracture was also tested and analysed by scanning electron microscopy.

## 3. Results and Discussions

### 3.1. Macroscopic Appearance and Dimensions

**Figure 3:**
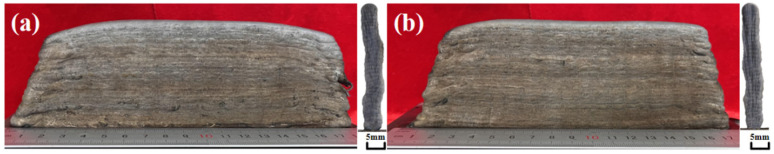
Complete appearance of specimens prepared by both processes. (**a**) Macroscopic morphology and cross-section of DC-deposited specimen and (**b**) macroscopic morphology and cross-section of pulsed DC-deposited specimen.

Figure 3a,b illustrate the appearance of the formed specimens under the two processes of DC deposition and pulsed DC deposition, respectively. The overall weld paths of the components under the two processes were smooth and flat, and no defects such as cracks and porosity were found on the surface and cross-section. However, both the DC deposition specimen and the pulsed DC deposition specimen collapsed at both ends. The collapse is more pronounced on the left side of the DC deposition specimen, which is due to the greater heat input. Tests show that the DC deposition specimen and pulsed DC deposition specimen were 55 mm and 50 mm in height and 5.57 mm and 5.43 mm in width, respectively. The height and width of the multi-layer deposition weld channel of the component are always kept constant, with absolute errors of 0.0593 mm and 0.0012 mm, respectively, which prove that the overall forming process is stable and the precision is reliable. When the number of deposited layers is equal, the height-to-width ratio of the welding channel of the pulsed DC-deposited parts is relatively low. The average layer thicknesses of single passes of DC deposition and pulsed DC deposition are 1.57 mm and 1.43 mm, respectively, indicating that the deposited layer becomes flatter after adding a pulse, which is conducive to maintaining the stability of the molten pool during the deposition process and improving the forming accuracy.

### 3.2. Microstructural Analysis

**Figure 4:**
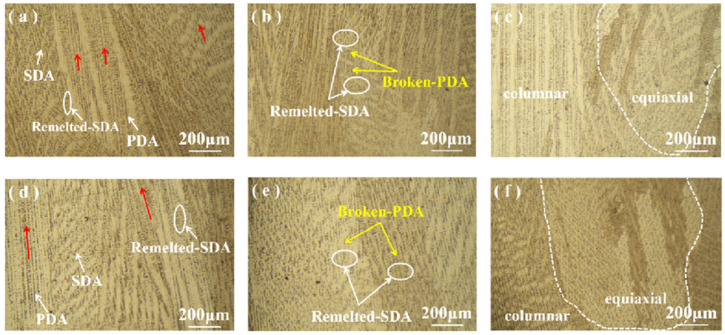
Comparison of optical microstructures of specimens from the two processes, with the red arrows representing the direction of columnar crystal growth and the dash lines delineates equiaxial crystal regions. (**a**) Bottom of DC deposition specimen; (**b**) middle of DC deposition specimen; (**c**) top of DC deposition specimen; (**d**) bottom of pulsed DC deposition specimen; (**e**) middle of pulsed DC deposition specimen; and (**f**) top of pulsed DC deposition specimen.

Figure 4 illustrates the comparison of the optical microstructures of different regions of the specimens perpendicular to the cross-section in the scanning direction for both DC and pulsed DC processes. Figure 4a–c show the grain morphology of the bottom, middle, and top regions of the GH4169 high-temperature alloy specimens prepared by DC deposition, respectively. Figure 4d–f show the grain morphology of the bottom, middle, and top regions of the GH4169 high-temperature alloy specimens prepared by pulsed DC deposition, respectively. Under the two process conditions, there are obvious differences in the microstructures of the materials between different deposited layers.

It is shown that the heat accumulation effect during metal additive manufacturing is the main reason for the microstructural transformation of multilayer deposited components [25]. A substantial quantity of columnar dendrites, many fine secondary dendrite arms (SDA), remelted dendrite arms, and primary dendrite arms (PDA) are seen in the bottom region of the specimens of both processes. In the middle region, a large number of cellular crystals are seen, and a profusion of broken primary dendrite arms and remelted secondary dendrite arms are clearly observed between the cellular crystals. The microstructure of the top region shows equiaxed crystals because there is no remelting of the subsequently deposited layers after the top deposition is completed. The average spacing of the nascent dendrite arms at the bottom of the DC-deposited specimen is 12.63 µm, and the growth directions of the columnar dendrites are not parallel. There is a slight variation in the lateral region, as shown by the red arrows in Figure 4a. The average spacing of the central primary dendrite arms is 17.66 µm. The top grain structure changes from columnar or cellular crystals to equiaxed dendrites. The PDA spacing of the primary dendrite arms slightly decreased compared with the middle region, with an average spacing of 30.9 µm. The average spacing of the primary dendrite arms at the bottom of the pulsed DC deposition specimen is 28.81 µm, and the growth direction of the columnar dendrites was parallel, as shown by the red arrows in Figure 4d. The average spacing of incipient dendrite arms in the middle is 16.48 µm. The grain structure at the top is transformed from columnar crystals or cellular crystals to equiaxed dendrites. The PDA spacing of primary dendrite arms is slightly decreased compared with the middle region, with an average of 11.66 µm. It can be seen that the average primary dendrite spacing gradually increased with the increase of deposition height, and there are finer dendrites. This is because during the cooling process of TIG additively manufactured GH4169 high-temperature alloy specimens, the closer the lower deposition layer is, the faster its cooling rate is, so the direction of grain growth is less fixed, and the dendrite spacing is smaller. The closer to the upper part of the deposition layer, the slower the cooling rate is under the action of the thermal accumulation effect, and the more fixed the direction of dendrite growth, the greater the intergranular distance.

**Figure 5:**
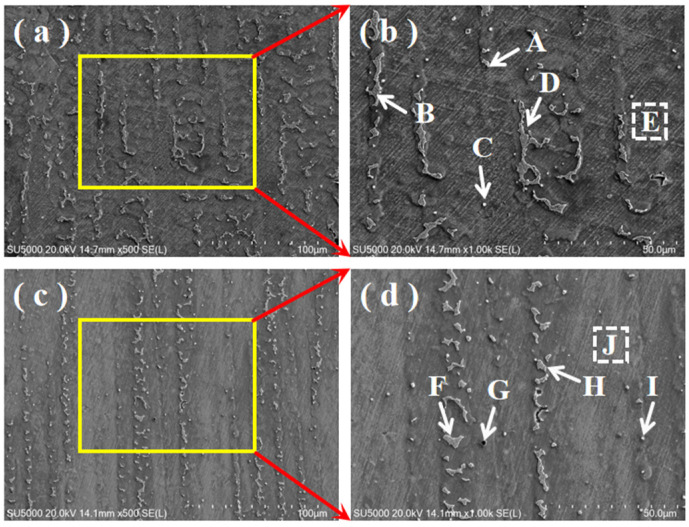
SEM images of the specimen microstructure, yellow frames and red arrows represent the magnified area and the letters define the scanning points. (**a**) Low magnification structure of DC-deposited specimen; (**b**) high magnification structure of DC-deposited specimen; (**c**) low magnification structure of pulsed DC-deposited specimen; and (**d**) high magnification structure of pulsed DC-deposited specimen.

The microstructures of DC and pulsed DC TIG additively manufactured GH4169 high-temperature alloy specimens at different locations are shown in Figure 5.

The microstructure of DC TIG that additively fabricated the GH4169 high-temperature alloy specimen by 500× scanning electron microscope is given in Figure 5a, which shows a number of precipitated phases with different morphologies in the dendritic zone. The microstructure of DC TIG that additively fabricated the GH4169 high-temperature alloy specimen by 1000× scanning electron microscope is given in Figure 5b. In order to determine the precipitation phases of the elements in the DC TIG additively fabricated GH4169 high-temperature alloy specimen, EDS elemental point scanning was carried out in the region, and the results are shown in Table 4.

It can be inferred from the EDS elemental point scan results that the precipitates in the DC TIG additively manufactured GH4169 high-temperature alloy specimen mainly consists of γ + Laves eutectic (Spectrum A), Laves massive (Spectrum B and D), and MC carbide particles enriched with Nb and Ti (Spectrum C), and the γ-matrix (Spectrum E) was detected. This result is similar to that of laser or electron beam additive manufacturing found in the literature [26,27,28]. The microstructure of the pulsed DC TIG additively fabricated GH4169 high-temperature alloy specimen by 500× scanning electron microscopy is given in Figure 5c. There are also many precipitated phases with different morphologies in the dendritic zone. The microstructure of pulsed DC TIG additively fabricated GH4169 high-temperature alloy specimen by 1000× scanning electron microscope is given in Figure 5d. In order to determine the precipitation phases of the elements in the pulsed DC TIG additively fabricated GH4169 high-temperature alloy specimen, EDS elemental point scanning was carried out in the region, and the results are shown in Table 5. It can be inferred from the EDS elemental point scan results that the precipitates in the pulsed DC TIG additively manufactured GH4169 high-temperature alloy specimen mainly consists of γ + Laves eutectic (Spectrum H), Laves massive (Spectrum F), Laves granular (Spectrum I), and Nb- and Ti-rich MC carbide particles (Spectrum G), and the γ-matrix (Spectrum J) was detected.

**Figure 6:**
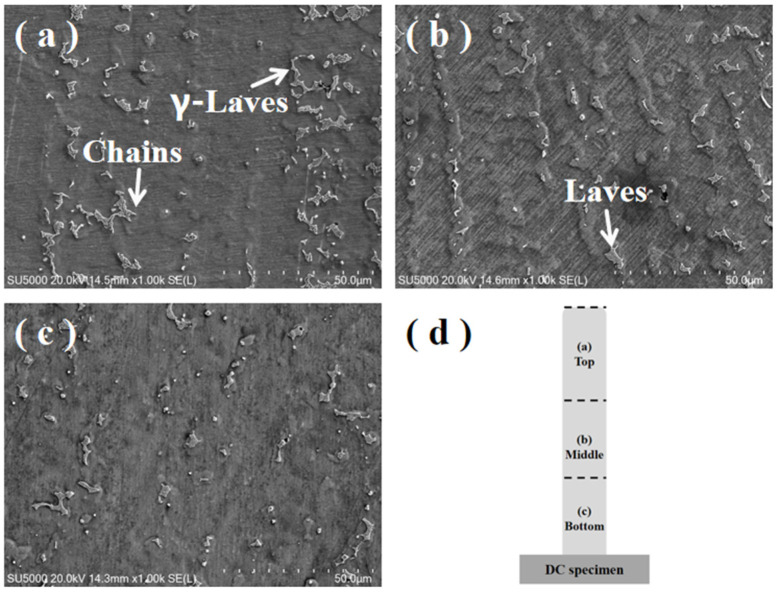
1000× SEM images of the microstructure of the DC-deposited specimen. (**a**) Top; (**b**) middle; (**c**) bottom; and (**d**) regional distribution map.

Figure 6 shows a 1000× SEM image of the microstructure of the DC-deposited specimen. Figure 6a–c show the precipitated phases in the top, middle, and bottom regions of the DC-deposited specimen, respectively. It can be clearly observed that there are many Laves phases interconnected with each other in the form of chains at the top. The Laves phases appearing in the middle area become rougher. Many fine lumpy or granular Laves phases are present at the bottom. These Laves phases are uniformly distributed in the columnar crystal region. The average diameters of the Laves phases shown in the figure were counted using the Image-Pro Plus software. The results show that the average diameter of the Laves phase in the microstructure of the DC TIG additively manufactured GH4169 high-temperature alloy specimen is 31.65 µm, and the difference between the average diameters of the largest and smallest Laves phase is 42.94 µm.

**Figure 7:**
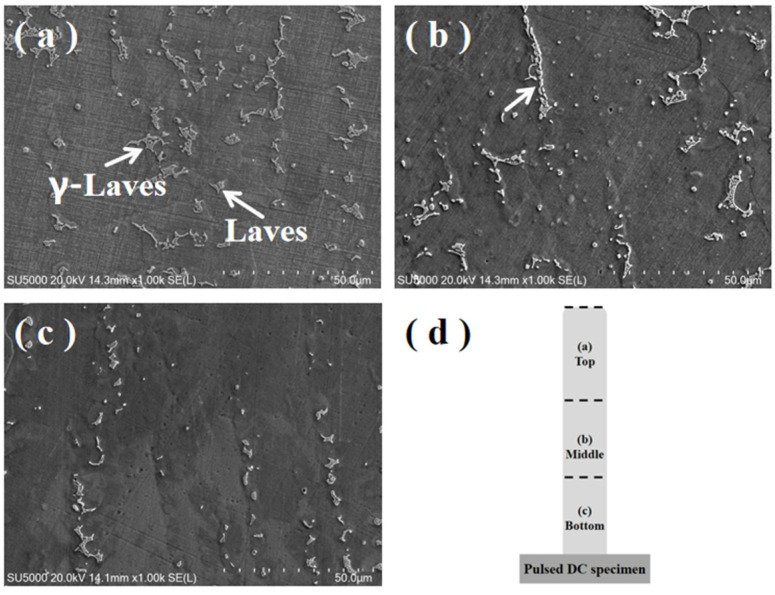
1000× SEM images of the microstructure of the pulsed DC-deposited specimen. (**a**) top; (**b**) middle; (**c**) bottom; (**d**) area distribution map.

Figure 7 shows 1000× SEM images of the microstructure of the DC-deposited specimen. Figure 7a–c demonstrate the precipitated phases in the top, middle, and bottom regions of the pulsed DC-deposited specimen, respectively. Compared with the DC TIG additively fabricated GH4169 high-temperature alloy specimen, the Laves phase of the pulsed DC specimen is significantly reduced. A small amount of γ + Laves eutectic and Laves phase exists at the top of the pulsed deposition specimen, the Laves phases appearing in the middle are interconnected into chains, and a small amount of fine lumpy or granular Laves phase exists at the bottom. The average diameters of the Laves phases shown in the figure were counted using the Image-Pro Plus software. The results show that the average diameter of the Laves phase in the microstructure of the pulsed DC TIG additively fabricated GH4169 high-temperature alloy specimen is 20.43 µm, and the difference between the average diameters of the largest and smallest Laves phases is 31.77 µm.

**Figure 8:**
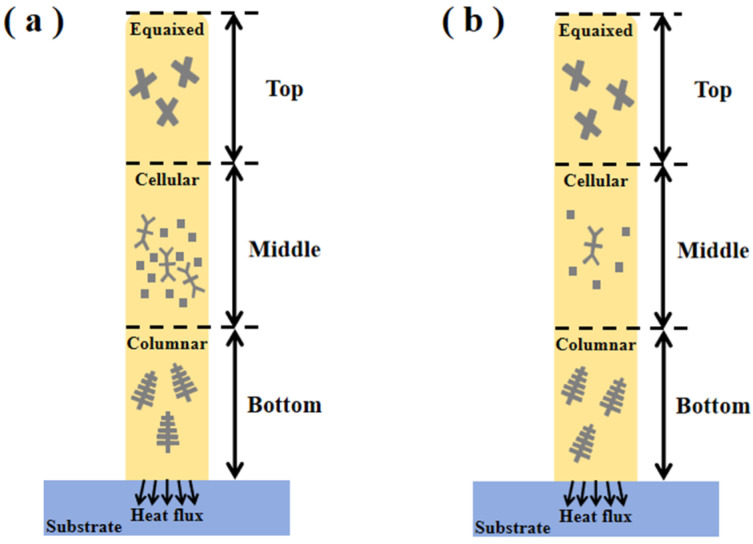
Schematic diagram of dendrite growth in specimens deposited by two processes. (**a**) DC deposition and (**b**) pulsed DC deposition.

The microstructure of the material between the different deposited layers was significantly different under the two process conditions. Schematic diagrams of dendrite growth of DC and pulsed DC TIG additively manufactured specimens are shown in Figure 8. The microstructures of the deposition-formed specimens from the bottom to the top show columnar crystals, cellular crystals, and equiaxed crystals, in this order. The bottom columnar crystals run through the fusion cladding in the deposition direction due to the epitaxial growth of remelted dendrites in the previous deposition layer. The growth direction of the columnar crystals at the bottom of the DC-deposited specimen is not parallel. On the contrary, the columnar crystals at the bottom of the pulsed DC deposition specimen grow in parallel directions. The number of Laves phases in the middle of the DC deposition specimen is more than that of the pulsed DC deposition specimen, as shown in Figure 8a,b. Laves phases are formed due to the microscopic segregation of Nb and Mo elements during the solidification of the alloys [29]. Laves phases are usually considered brittle phases, which are detrimental to the tensile, fatigue, and creep properties of the materials, leading to deterioration of the mechanical properties [30]. This is the main reason for the superior mechanical properties of the pulsed DC-deposited specimens than the DC-deposited specimens. The microstructure of the top layer shows equiaxed crystals because there is no remelting of the subsequently deposited layers after the deposition of the top layer is completed. The change in the temperature gradient in the top layer causes a change in the growth direction of the dendrite spindle, thus forming an equiaxed crystal at the top.

**Figure 9:**
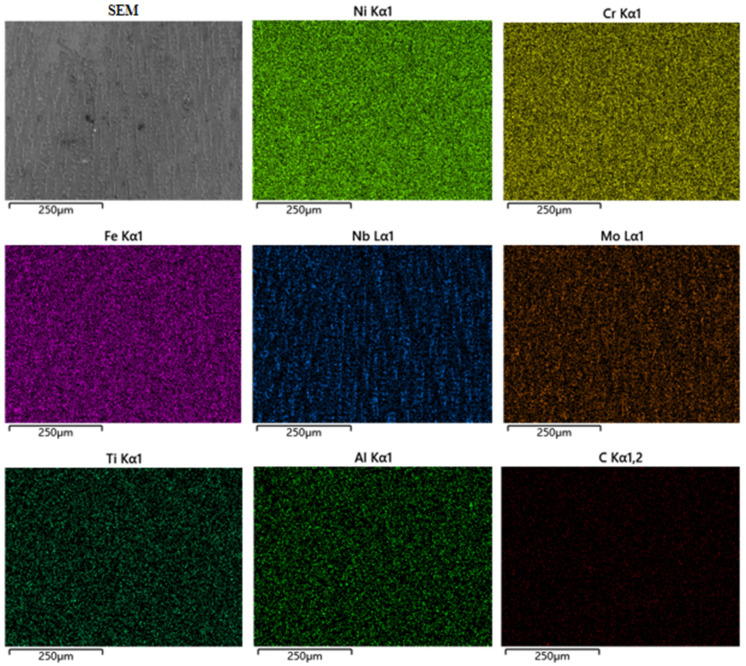
EDS surface scanning of the GH4169 high-temperature alloy specimen fabricated by DC TIG additive manufacturing.

**Figure 10:**
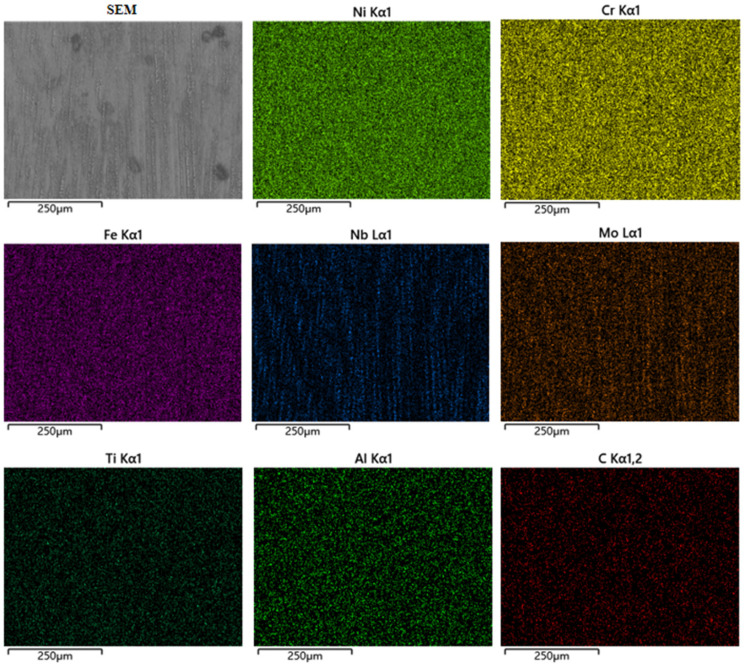
EDS surface scanning of the GH4169 high-temperature alloy specimens fabricated by pulsed DC TIG additive manufacturing.

The EDS surface scans of DC TIG additively fabricated GH4169 high-temperature alloy specimens and pulsed DC TIG additively fabricated GH4169 high-temperature alloy specimens are shown in Figure 9 and Figure 10 and Table 6. As shown in Table 6, the content of Ni element in DC-deposited specimen and pulsed DC-deposited specimen is 46.39% and 47.24%, respectively. The elemental C contents of DC deposition specimens and pulsed DC deposition specimens are 9.82% and 7.91%, respectively. The elemental C content of DC deposition specimens is higher than that of pulsed DC deposition specimens. Due to the high melting point and strength of MC carbide, it can play the role of skeleton strengthening in the alloy when the precipitation quantity is small [31]. However, if there is a C elemental bias, this can lead to severe segregation of carbide precipitation in the alloy. Higher carbide content in a region will lead to a decrease in tensile strength and microhardness in the corresponding region, which is very unfavourable for the performance of the GH4169 high-temperature alloy. The pulsed DC TIG additive manufacturing process for the GH4169 high-temperature alloy optimises this phenomenon.

**Figure 11:**
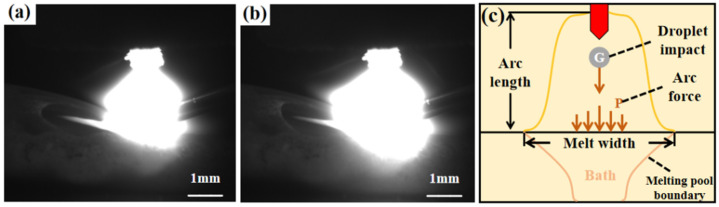
Arc morphology captured by a high-speed camera. (**a**) During DC deposition; (**b**) during pulsed DC deposition; and (**c**) force distribution map.

Figure 11a,b show the arc morphology under DC and pulsed DC processes captured by the high-speed camera system. In both processes, the electric arc not only converts electrical energy into heat but also melts the filler material and the base material. It is also a source of force, as the arc can generate mechanical force. The mechanical force P generated by the arc can be obtained from Equation (1), which shows that the arc force is proportional to the square of the current. Therefore, the greater the current, the greater the mechanical force generated by the arc [32].

(1) $$P=\frac{\mu_{0}\mu }{8\pi }\cdot I^{2}=KI^{2}$$

*µ*_0_ and *µ* are the permeability coefficients of air and arc atmosphere. *K* is the coefficient for different currents where *K* = (1.02~1.04) × 10^−6^ N/A^2^. A comparison of Figure 11a,b shows that when DC deposition is used, the wire forms a molten droplet mainly at the edge of the arc and then is electromagnetically fed into the molten pool. Due to the bias of the electromagnetic force on the droplets during this process, the droplets are susceptible to side-spinning and spattering before detaching from the wire. The transition mode of the droplet is mainly a large droplet transition, and the molten pool is also narrow and shallow. When pulsed DC deposition is used, the wire melts into the molten pool, mainly in the centre of the arc, and the electromagnetic force is more uniform. The directionality of the droplet transition is better, and the problem of spattering and swirling rarely occurs. The droplet transition mode is mainly a fine droplet transition. The arc stiffness increases during pulsed DC deposition. Under the arc pressure, the molten pool becomes wide and flat with a relatively stable morphology, which is more favourable to the continuous deposition of metal. This indicates that the introduction of the pulse effectively improves the precision of droplet transition and the stability of the melt pool, which improves the forming performance and process stability of the components.

### 3.3. Mechanical Properties

#### 3.3.1. Microhardness

**Figure 12:**
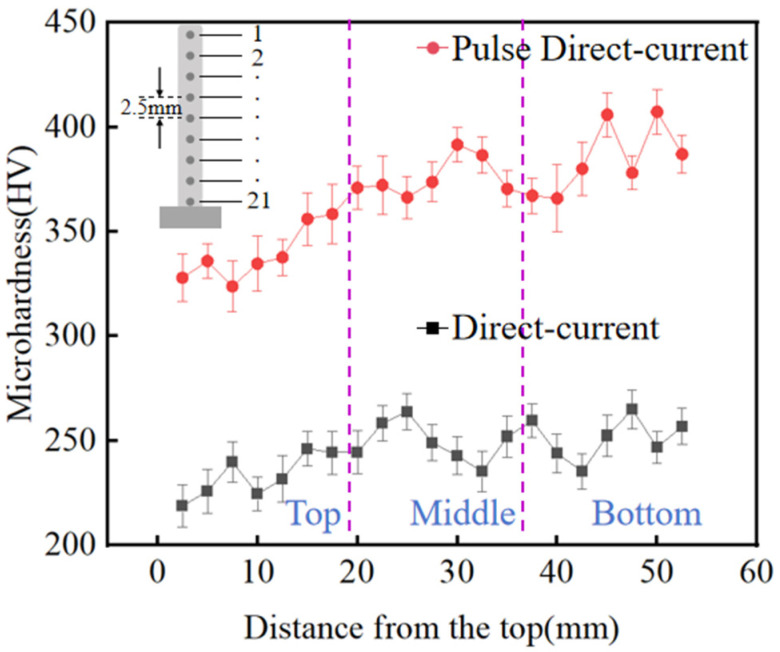
Curve of Micro-Vickers hardness versus apical distance, the dotted lines separate the different areas.

Microhardness tests were conducted on DC TIG additively manufactured GH4169 high-temperature alloy specimens and pulsed DC TIG additively manufactured GH4169 high-temperature alloy specimens. Figure 12 shows the variation curves of Micro-Vickers hardness versus apical distance. The results show that the microhardness of both DC-deposited specimens and pulsed DC-deposited specimens fluctuated significantly. The microhardness of the pulsed DC deposition specimen changed significantly compared to the DC deposition specimen, and there was a significant increase in all parts of the specimen. The average microhardness of the bottom of the DC deposition specimen was 251.5 HV, the average microhardness of the middle was 249.5 HV, the average microhardness of the top was 233.1 HV, and the overall average microhardness was 244.7 HV. The average microhardness of the lower part of the pulsed DC deposition specimen was 384.5 HV, the average microhardness of the middle was 376.0 HV, the average microhardness of the top was 339.2 HV, and the overall average hardness was 366.6 HV. Compared with the DC deposition specimens, the average microhardness of the bottom of the pulsed DC deposition specimens increased to 106.1 HV, the average microhardness of the middle increased to 126.5 HV, the average microhardness of the top increased to 133.0 HV, and the overall average microhardness increased to 121.9 HV. The microhardness showed a gradual increase with the increase of the distance from the top. There are two main reasons for this change. On the one hand, the grain size and spacing of the additive specimens increase with the increase of the specimen height. On the other hand, the number of precipitated phases gradually increases with the increase of the specimen height, and the solid solution strengthening elements in the matrix decrease. The microhardness of the pulsed DC deposition specimens is higher than that of the DC deposition specimens, mainly because the microstructure of the pulsed DC deposition specimens is transformed from columnar crystals to fine cellular crystals, and the grain spacing, as well as the size and number of precipitated phases, show a tendency to decrease, so the microhardness of the pulsed DC deposition specimens increases.

#### 3.3.2. Tensile Properties

**Figure 13:**
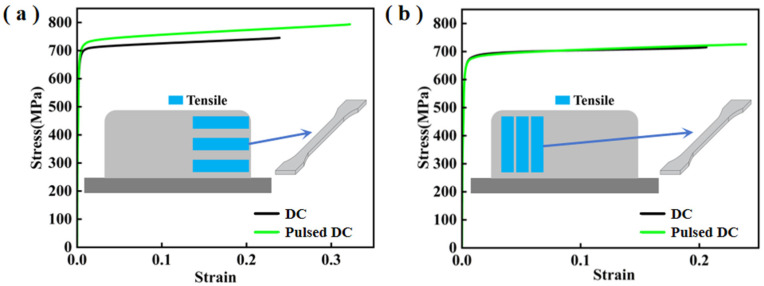
Stress-strain curves of DC and pulsed DC specimens at room temperature. (**a**) Horizontal direction and (**b**) vertical direction.

Table 7 shows the comparison of the tensile properties of DC-deposited specimens and pulsed DC-deposited specimens in both horizontal and vertical directions. Figure 13 shows the stress-strain curves of DC-deposited specimens and pulsed-deposited specimens in both horizontal and vertical directions. The results show that the TIG additively fabricated GH4169 specimens have obvious anisotropy. The average tensile strengths of DC-deposited and pulsed DC-deposited specimens in the horizontal direction were 745.00 MPa and 792.47 MPa, and the elongation was 23.89% and 32.21%, respectively. In the vertical direction, the average tensile strength of DC-deposited specimens and pulsed DC-deposited specimens were 714.93 MPa and 725.30 MPa, and the elongation was 20.66% and 23.94%, respectively. It can be seen that the microstructural inhomogeneity within and between the deposited layers of the metal results in significantly higher strength and plasticity along the horizontal direction of metal deposition than the corresponding indexes in the vertical direction. Meanwhile, the strength and plasticity of the components obtained from the optimised pulsed DC TIG additive manufacturing process are significantly improved compared with that of the DC TIG additive manufacturing process, which is conducive to further improving the performance of the finished additive components.

### 3.4. Fracture Morphology

**Figure 14:**
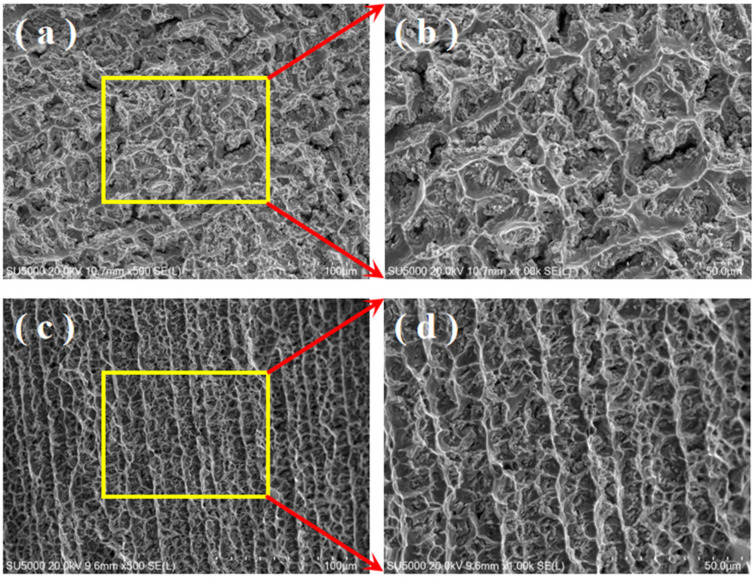
SEM of tensile fracture, yellow frames and red arrows represent the magnified area. (**a**) Tensile fracture micro-morphology of DC-deposited specimen (500×); (**b**) tensile fracture micro-morphology of DC-deposited specimen (1000×); (**c**) tensile fracture micro-morphology of pulsed DC-deposited specimen (500×); and (**d**) tensile fracture micro-morphology of pulsed DC-deposited specimen (1000×).

Figure 14 shows the micro-morphology of tensile fracture of the GH4169 high-temperature alloy specimen fabricated by DC TIG additive manufacturing and the GH4169 high-temperature alloy specimen fabricated by pulsed DC TIG additive manufacturing. Figure 14a shows the fracture morphology of the low-fold DC-deposited specimen, which shows a large surface of tough nests and is exhibited through a crystal brittle damage mode. Figure 14b illustrates the fracture morphology of the high-fold DC-deposited specimen, where many large and deep micropores are arranged along the boundaries so that the PDA can be clearly observed. Figure 14c illustrates the fracture morphology of the low-fold pulsed DC-deposited specimen, from which the grain boundaries are seen to become blurred. Figure 14d illustrates the fracture morphology of the specimen deposited by high-fold pulsed DC, and a large number of finer tough nests can be observed, which indicates a ductile damage mode.

## 4. Conclusions

- The overall forming process of the components was relatively stable under DC TIG additive manufacturing and pulsed DC TIG additive manufacturing processes. In the reciprocating deposition process, the average layer thicknesses were 1.57 mm and 1.43 mm, respectively, and the depth-to-width ratios of the weld channels of the pulsed DC deposition specimens were relatively low. The deposited layer became flatter after adding the pulse, which was conducive to maintaining the stability of the molten pool during the deposition process and improving the forming accuracy.
- The microstructure distribution of the sedimentary layer from bottom to top was relatively heterogeneous. The bottom layer was columnar dendrites, the middle layer was cellular crystals, and the top layer was equiaxed crystals. Compared with the DC TIG additive manufacturing of the GH4169 high-temperature alloy specimens, the Laves phase of the pulsed DC specimens was significantly reduced, which improved the plasticity and toughness of the material.
- The carbon content of the DC deposition specimen was higher than that of the pulsed DC deposition specimen. Higher carbide content in a certain region will lead to a decrease in tensile strength and microhardness in the corresponding region, which is very unfavourable to the properties of the GH4169 high-temperature alloy. The pulsed DC TIG additive manufacturing process for the GH4169 high-temperature alloy optimised this phenomenon.
- The droplet transition modes of the DC and pulsed DC deposition samples were large droplet transition and fine droplet transition, respectively. The fine-drop transition mode under pulsed conditions had better directivity and less splashing, which was more conducive to ensuring the forming accuracy and process stability of the components.
- Both the DC-deposited specimens and the pulsed DC-deposited specimens showed significant anisotropy in the overall mechanical properties. The strengths in the horizontal direction were higher than those in the vertical direction by 30.07 MPa and 67.17 MPa, respectively. This apparent property inhomogeneity affected the performance of the components.
- The fracture morphology of the DC-deposited specimen showed a large surface of ligament foci and exhibited a crystal penetrating ductile damage mode. The fracture morphology of the pulsed DC-deposited specimen, where a large number of finer brittle nests could be observed, indicated a ductile damage mode.

## Figures and Tables

**Table 1:** Q235 chemical composition (wt.%).

C	Mn	Si	S	P	Fe
0.14–0.22	0.30–0.65	≤0.30	≤0.050	≤0.045	Balance

**Table 2:** Chemical composition of GH4169 high-temperature alloy wire (wt.%).

C	Cr	Mo	Ni	Nb	Ti	Al	Si	Mn	P	S
0.037	19.5	3.11	52.9	5.16	0.88	0.38	0.10	0.10	0.005	0.003

**Table 3:** GH4169 high-temperature alloy TIG additive manufacturing process parameters.

Specimen	Wire Feed Speed V_f_/(cm/min)	Welding Speed V_h_/(mm/min)	Average Current I/A	Peak Current I/A	Base Value Current I/A	Duty Cycle (%)	Pulse Frequency (HZ)
A (DC)	350	300	250	-	-	-	-
B (Pulsed DC)	350	300	250	294	206	50	300

**Table 4:** EDS elemental point scan results of DC-deposited specimens.

Element (wt.%)	C	Al	Ti	Cr	Fe	Ni	Nb	Mo
Spectrum A	0.00	0.16	0.82	19.46	19.46	47.47	10.25	4.87
Spectrum B	0.00	0.21	1.50	10.53	19.35	46.38	18.08	3.95
Spectrum C	12.74	0.45	1.43	18.18	14.03	45.31	5.22	2.63
Spectrum D	0.00	0.33	1.57	11.68	16.78	43.69	22.5	3.45
Spectrum E	1.09	0.49	0.66	19.05	17.44	55.37	3.05	2.84

**Table 5:** EDS elemental point scan results of pulsed DC-deposited specimens.

Element (wt.%)	C	Al	Ti	Cr	Fe	Ni	Nb	Mo
Spectrum F	0.00	0.57	1.04	18.63	18.08	47.05	11.15	3.48
Spectrum G	16.51	0.81	1.96	12.31	12.76	45.07	5.50	5.07
Spectrum H	0.00	1.13	1.13	16.11	13.77	48.78	15.54	3.54
Spectrum I	0.00	0.25	1.34	13.14	10.94	49.05	20.55	4.74
Spectrum J	0.89	0.54	0.60	19.12	17.86	55.72	2.51	2.77

**Table 6:** EDS surface scanning elemental content of DC-deposited and pulsed DC-deposited specimens.

Element (wt.%)	C	Al	Ti	Cr	Fe	Ni	Nb	Mo
A (DC)	9.82	0.45	0.85	18.19	16.35	46.39	4.9	3.04
B (Pulsed DC)	7.91	0.46	0.85	18.69	16.73	47.24	4.99	3.14

**Table 7:** Comparison of tensile properties of DC-deposited specimen with pulsed DC-deposited specimen.

Specimen	Horizontal Direction	Horizontal Direction	Vertical Direction	Vertical Direction
	Tensile Strength	Elongation	Tensile Strength	Elongation
	(MPa)	(%)	(MPa)	(%)
A (DC)	745	23.89	714.93	20.66
B (Pulsed DC)	792.47	32.21	725.3	23.94

## Data Availability

The original contributions presented in the study are included in the article, further inquiries can be directed to the corresponding author.
